# Sacrococcygeal teratoma: evaluation of its approach, treatment and follow-up in two reference children cancer centers in Brazil / Rio de Janeiro

**DOI:** 10.1590/0100-6991e-20223341-en

**Published:** 2022-08-25

**Authors:** VANESSA DO NASCIMENTO SANTOS, SIMONE DE OLIVEIRA COELHO, ALAN ARAUJO VIEIRA

**Affiliations:** 1 - Universidade Federal Fluminense, Saúde Materno-Infantil - Rio de Janeiro - RJ - Brasil; 2 - Instituto Nacional do Câncer, Cirurgia Pediátrica Oncológica - Rio de Janeiro - RJ - Brasil

**Keywords:** Teratoma, Neoplasms, Neoplasms, Germ Cell and Embryonal, Teratoma, Neoplasias, Neoplasias Embrionárias de Células Germinativas

## Abstract

**Introduction:**

sacrococcygeal teratoma (TSC) is the most common tumor of the neonatal period. Alphafetoprotein is an important tumor marker and is used in the follow-up period as a marker of malignancy. The complete surgical resection of the tumor associated with coccygectomy is the standard treatment and chemotherapy in different stages are necessary. Follow-up consists of serial exam: tumor markers, imaging searching to possible metastasis sites, in addition to a complete physical examination.

**Methodology:**

a descriptive, retrospective, study was carried out by analyzing a chart of 25 patients of two different reference children cancer center; with TSC in the State of Rio de Janeiro from 2004 to 2019. The clinical and epidemiological data collected were described and a comparison was made between these two centers studied.

**Results:**

the sociodemographic characteristics found were similar to those described in the medical literature. Data related to treatment and follow-up, such as the use of chemotherapy, use of specific imaging tests, digital rectal examination, and outpatient follow-up, differed between the two centers studied. There was a 25% loss of follow-up.

**Conclusion:**

the characteristic of being a non-cancer center can interfere with the full application of the current protocol for the treatment of sacrococcygeal teratoma. The knowledge of the data of the studied cases can allow the optimization of the approach of the patients with this pathology and generate discussions about the integral application of the specific therapeutic protocol in the medical centers that are qualified for such treatment.

## RESUMO

**Introdução:**

teratoma sacrococcígeo é o tumor neonatal mais comum. Alfafetoproteína é um marcador tumoral importante e é utilizado no período de seguimento como um marcador de malignidade. A ressecção cirúrgica completa do tumor associado a coccigectomia é o tratamento padrão, associado a quimioterapia em determinados estadiamentos. Exames de seguimento consistem em avaliação de marcadores tumorais, investigação de sítios de metástases, além de um completo exame físico.

**Metodologia:**

foi realizado um estudo retrospectivo descritivo, através da análise de 25 pacientes em dois centros de referência em tratamento oncológico infantil, com teratoma sacrococcígeo no Estado do Rio de Janeiro, entre 2004 e 2019. Os dados clínicos e epidemiológicos foram descritos, comparando-se os dois centros.

**Resultados:**

as características socio-demográficas foram similares ao descrito na literatura médica. Os dados relativos ao tratamento e seguimento, como o uso de quimioterapia, uso de testes de imagem específicos, exame de toque e retal e seguimento ambulatorial, foi diferente entre os dois centros. Tivemos uma perda de seguimento de 25%.

**Conclusão:**

a característica de ser um centro não oncológico pode interferir com a aplicação do protocolo de tratamento de teratoma sacrococcígeo. O conhecimento dos dados estudados pode permitir a otimização da abordagem dos pacientes com esta patologia e gerar discussões sobre a aplicação integral dos protocolos terapêuticos em centros médicos que são qualificados para tal tratamento.

## INTRODUCTION

Sacrococcygeal teratoma (SCT) is the most common neoplasm of the neonatal period, with an incidence of approximately 1:40,000 live births^1^ ^,^ ^2^. It corresponds to 70% of all teratomas of childhood, being predominant in females, with a ratio of 4 girls to every boy^3^ ^,^ ^4^.

About 20% of SCT are malignant at diagnosis and 5% are metastatic. When diagnosed before the age of 6 months, they are rarely malignant (2%), whereas teratomas diagnosed after the sixth month of life have a malignancy rate around 65%^5^ ^-^ ^7^.

The most commonly used classification for SCT is the one developed by Altman^6^, which is based on the anatomical presentation of the lesion, such as the exophytic and/or intrapelvic extent of the tumor. 

In Brazil, the TCG/GALOP Protocol (2017) is used by cancer centers to guide the diagnostic approach, treatment, and follow-up of patients with SCT. However, fetal medicine and obstetric high-risk referral centers, responsible for receiving most patients with Type I SCT, usually diagnosed still in the prenatal period, do not routinely follow this referral protocol, which can directly influence the prognosis of these patients.

The objective of this research is to analyze the epidemiological, clinical and laboratory data of patients with sacrococcygeal teratoma surgically treated in the two main public referral centers for this pathology in the state of Rio de Janeiro, in the last 16 years. This study was approved by the Research Ethics Committee of the Institutions mentioned.

## METHODOLOGY

Observational, retrospective study, with the analysis of medical records of patients diagnosed with SCT surgically treated from 2004 to 2019, in the two main reference centers for this tumor in the state of Rio de Janeiro.

Data from all patients with a pre-operative diagnosis of SCT who underwent surgery for tumor resection were included in this study. Data from patients who had no histological confirmation of the diagnosis were excluded.

Data collected included gender, race, place of birth, prenatal or postnatal diagnosis, results of tests used for diagnostic confirmation, histopathological report, serum tumor marker levels, the presence of early or late surgical complications, frequency of recurrence, postoperative sequelae and deaths, and the maintenance of outpatient follow-up. Patients who had already been discharged from the outpatient clinic were contacted by telephone, in order to obtain updated information of all cases.

The data were analyzed for relative and absolute frequency and compared by the chi-squared test with Fischer’s correction, when necessary. Continuous variables were summarized by measures of central tendency and compared by Student’s t-test, or by non-parametric test when with non-normal distribution. SPSS16.0 statistical package was used. The study was designed according to the guidelines and norms regulating research involving human beings and approved by the Research Ethics Committee of the two participating centers.

## RESULTS

From a total of 31 suspected cases selected, 6 were excluded because they did not histologically confirm the diagnosis of SCT. 

Most patients included in the study were female, white, and had not been born in Rio de Janeiro city. At the time of surgical treatment in the reference center for fetal medicine and high obstetric risk, the mean age of patients was 97 days, and in the medical oncology center was 324 days (p=0.063). It is important to mention that all patients treated at the oncology center had their diagnosis after birth (Table 1). In this center, tomography and/or magnetic resonance imaging were the most used preoperative screenings, and the majority of these patients were classified as type 4, according to Altman. The SCT stage was not reported in 100% of the patients seen at the reference center for fetal medicine and high obstetric risk; in addition, there were no cases of biopsy procedures performed (Table 1).

**Table 1:** Socio-demographic and clinical data of patients diagnosed with sacrococcygeal teratoma treated surgically from 2004 to 2019, compared by reference center.

Variables		Reference center for fetal and obstetric risk n=18 n(%)	Reference Cancer Center n=7 n(%)	p-value
Gender	Female	14 (78.0%)	7 (100.0%)	0.450
Race	White	11 (61.0%)	5 (71.4%)	
	Black	2 (11.0%)	1 (14.3%)	
	Brown	5 (28.0%)	1 (14.3%)	
Variables		Reference center for fetal and obstetric risk n=18 n(%)	Reference Cancer Center n=7 n(%)	p-value
Origin	Born at the Origin Hospital	8 (44.0%)	0 (0.0%)	0.097
	Patients Referred from other origins	10 (56.0%)	7 (100.0%)	
Location	Rio de Janeiro (Capital)	8 (44.0%)	3 (42.9%)	0.708
	Others	10 (56.0%)	4 (57.1%)	
Diagnostic	Before birth	10 (56.0%)	0 (0.0%)	0.775
	After birth	8 (44.0%)	7 (100.0%)	
Other Diagnostics	Yes^1,2^		2 (28.6%)	0.096
Pre-Operative Tests	CT	13 (72.2%)	0 (0.0%)	<0.001
	CT + MR	1 (5.6%)	5 (71.4%)	
	CT + USG	3 (16.6%)	0 (0.0%)	
	MR + USG	1 (5.6%)	0 (0.0%)	
	MR	0 (0.0%)	2 (28.6%)	
Altman Classification	Type I	11 (61.1%)	0 (0.0%)	0.042
	Type II	1 (5.6%)	1 (14.3%)	
	Type III	2 (11.1%)	1 (14.3%)	
	Type IV	4 (22.2%)	5 (71.4%)	
Staging	Stage I	0 (0.0%)	2 (28.6%)	<0.001
	Stage II	0 (0.0%)	1 (14.2%)	
	Stage III	0 (0.0%)	2 (28.6%)	
	Stage IV	0 (0.0%)	2 (28.6%)	
	Not Informed	18 (100.0%)	0 (0.0%)	
Biopsy	Yes	0 (0.0%)	3 (42.9%)	0.023
Alpha-fetoprotein (AFP)	No	11 (61.1%)	2 (28.6%)	0.214
	Altered	6 (33.3%)	5 (71.4%)	
	Not Informed	1 (5.6%)	0 (0.0%)	
Beta-HCG (BHCG)	Not Altered	11 (61.1%)	7 (100.0%)	0.151
	Altered	0 (0.0%)	0 (0.0%)	
	Not Informed	7 (38.9%)	0 (0.0%)	
Lactate Dehydrogenase (LDH)	Not Altered	1 (5.6%)	3 (42.9%)	
	Altered	5 (27.8%)	4 (57.1%)	0.005
	Not Informed	12 (66.6%)	0 (0.0%)	
Neoadjuvant	Yes			<0.001

CT: computed tomography; MR: magnetic resonance; USG: ultrasound.

Alpha-fetoprotein (AFP) was checked in all patients under follow-up in the oncology center, and in most of those treated in the fetal medicine and obstetric high-risk center; however, in this center, B-HCG dosage was not performed or reported in the medical record of 39% of patients, and LDH dosage in 66% (p=0.005) of them (Table 1). 

As for surgical treatment, in most situations the perineal approach by Chevron incision was used, and in all cases, a complete resection of the coccyx was recorded. None of the patients seen at the high-risk fetal and obstetric medicine center underwent adjuvant chemotherapy (Table 1).

In seventy-one percent of SCT admitted to the oncology center and in only 22% of those admitted to the high-risk fetal and obstetric medicine center (p=0.007) a histological proof of malignancy was found (Table 2). The most common histopathological reports were mature teratoma (60%) and endodermal sinus tumor (36%), all in girls; only 1 case of immature teratoma was described. 

**Table 2:** Data regarding treatment and follow-up of patients diagnosed with sacrococcygeal teratoma treated surgically from 2004 to 2019, compared by reference center.

Variables		Reference center for fetal and obstetric risk n=18 n(%)	Reference cancer center n=7 n(%)	p-value
Surgical approach	Perineal	17 (94.4%)	6 (85.7%)	0.920
	Abdominal-Perineal	1 (5.6%)	1 (14.3%)	
Histopathological Report	Mature Teratoma	13 (72.2%)	2 (28.6%)	0.007
	Immature Teratoma	1 (5.6%)	0 (0.0%)	
	Malignant Teratoma*	4 (22.2%)	5 (71.4%)	
Adjuvant Chemotherapy	Yes	0 (0.0%)	2 (28.6%)	0.123
Post-surgery Complications	No Complications	10 (55.6%)	2 (28.6%)	0.078
	Infection	0 (0.0%)	1 (14.3%)	
	Dehiscence)	4 (22.2%)	0 (0.0%)	
	Infection + Dehiscence	4 (22.2%)	4 (57.1%)	
Alpha-fetoprotein (AFP) Post-Surgery	Not Altered	15 (83.3%)	6 (85.7%)	0.647
	Altered	3 (16.7%)	1 (14.3%)	
Rectal exam	Normal	5 (27.8%)	7 (100.0%)	0.005
	Not Performed	13 (72.2%)	0 (0.0%)	
Post-surgery Tests	USG	12 (66.6%)	3 (42.9%)	0.004
	CT	3 (16.7%)	0 (0.0%)	
	CT + USG	0 (0.0%)	4 (57.1%)	
	Not Performed	3 (16.7%)	0 (0.0%)	
Surgery Sequelae	Yes^1,2^	4 (22.2%)	2 (28.6%)	0.644
	No	12 (66.7%)	5 (71.4%)	
	No Information	2 (11.1%)	0 (0.0%)	
Outcome	Alive without disease	6 (33.3%)	4 (57.1%)	0.022
	Alive with disease	0 (0.0%)	1 (14.3%)	
	Referred to another hospital	1 (5.6%)	0 (0.0%)	
	Loss of follow-up	5 (27.8%)	0 (0.0%)	
	Outpatient discharge	6 (33.3%)	0 (0.0%)	
	Death**	0 (0.0%)	2 (28.6%)	

CT: computed tomography; MR: magnetic resonance; USG: ultrasound. ^1^Reference center for fetal and obstetric risk: Sacral Dimple (3); PilonidalCyst (1); Myelomeningocele (2); SpinaBifida (1). ^2^Reference cancer center: Sacral Dimple (1); Meningocele (1).

An examination of the rectum was not performed in 72% of the patients being followed up outside the oncology center; in addition, there was also a significant difference between the two centers regarding procedures performed for post-operative control (Table 2). 

The AFP presented high serum levels at postoperative follow-up in only 4 cases, and all of them had tumor recurrence. The percentage of postoperative sequelae was similar for the two centers. In most cases, there were no surgical sequelae; it was present in 24% of them. Most of the post-operative sequelae occurred as a result of infections and/or suture wound dehiscence. Long-term sequelae described were urinary incontinence or retention, and tumor recurrence; there were no reports of fecal retention. It was not possible to assess the presence of sequelae in 8% of cases due to loss of follow-up.

Death data are present only in the oncology center (28%), however, the high rate of loss to follow-up and non-outpatient follow-up (61%) in the non-cancer center may have prevented the analysis of these data. All patients treated at the oncology center remain under outpatient follow-up. Regarding the two deaths reported, one was directly related to the complications of Patau Syndrome.

## DISCUSSION

In this study, the main differences between the two centers were related to the clinical management of patients regarding diagnosis and treatment, as well as follow-up.

Socio-demographic aspects of the sample were similar to those in the literature. White individuals were the most affected, and the gender data showed a higher percentage of females, with a 5:1 ratio^6^ ^,^ ^8^ ^-^ ^10^.

A diagnosis of SCT during the prenatal period is the reality of most cases described in the literature, mainly reporting patients from developed countries^4^ ^,^ ^11^. Tumors classified as type I are the majority, due to the presence of an evident exophytic component, and can be usually visualized on routine obstetric ultrasonography^12^ ^,^ ^13^. However, in this study, postnatal diagnosis was performed in the majority of cases assessed. In the oncology center there were no cases of diagnosis made during the prenatal period, and even in the high-risk pregnancy follow-up center, only eight cases were diagnosed during the prenatal period. 

This may be evidence of inadequate prenatal care offered to this population. Regarding patients who were referred to the high-risk pregnancy center after birth, three different situations could be identified: no prenatal care; prenatal care without image scans; and cases where the fetal morphology was evaluated by image screenings with no visualization of the tumor. Most of these patients were referred from cities in outside the metropolitan region. The availability of adequate prenatal care to allow the suspicion and/or early diagnosis of cases of fetal malformation is still a challenge in Brazil.

As expected, in the oncology center there was a higher incidence of tumors classified as type 4 according to Altman’s criteria. These tumors were submitted to tests for staging, in addition to biopsy for diagnostic confirmation in doubtful cases, as directed by the protocol^5^. The histopathological diagnosis is fundamental to define the therapeutic conduct, mainly regarding the indication of neoadjuvant chemotherapy, in order to allow a safe surgery with no damage to adjacent structures. Surgeries requiring excision of vital organs in benign cases of SCT and mutilating surgeries in malignant cases should not be performed without prior chemotherapy treatment^14^.

Tumor staging was performed in only 28% of the studied cases. The fact that most cases of SCT diagnosed in the neonatal period are classified as Type I, almost always with histology considered benign and with minimal chance of long-term relapse, may have influenced this finding^15^ ^,^ ^16^. In most cases, newborns are considered to have localized disease. In this study, however, the occurrence of SCT classified in all staging levels (I to IV) highlights the importance of not ignoring the possibility of metastasis, even for those with Type I classification. Similarly, in addition to guiding the diagnosis, the measurement of tumor markers during the initial evaluation of all cases, as recommended by the protocol, can influence the indication of a neoadjuvant treatment with chemotherapy in specific cases. In the oncology center assessed, the evaluation of tumor markers was performed in all cases, which enabled the indication of neoadjuvant chemotherapy treatment in two patients. 

The abdominal-perineal surgical approach was performed only in two patients in this sample, both classified as Type IV SCT. The combined approach was used in cases where tumor resection was not feasible by a single approach; the tumor volume associated with the involvement of adjacent structures determined the need for an approach that allowed the safe resection of the lesion and the performance of a coccygectomy, an indispensable part of this surgery^16^.

Although coccygectomy was reported in all cases studied, the complete resection of the coccyx was not confirmed by image scans. The need for radiological confirmation of a complete coccygectomy has been discussed in the literature and is still not a consensus. The high cost of confirming a procedure that can be observed during surgery makes this indication questionable5. Information regarding the presence or absence of the coccyx was not found in histopathology reports. The confirmation of the presence of the coccyx by the pathologist, although not a common request, could be of great help; the inclusion of this information in the histopathological reports of SCT should be discussed and encouraged. 

Regarding postoperative sequelae, it is worth noting that the results were similar in both centers, even though the oncology center received the most complex cases. The main surgical complications were infection and surgical wound dehiscence, with percentages similar to those described in the literature^5^ ^,^ ^17^. These findings may be justified by the proximity of the surgical site to the anus, thus enabling contamination with colonic bacteria; in general, these complications are also associated with inadequate local hygiene and lack of systematization in the care of surgical dressings^18^ ^-^ ^20^. 

In the follow-up of patients with SCT, it is recommended the monitoring of serum levels of tumor markers, image scans and rectal examination. According to the TSC/GALOP 2017 protocol, this part of the physical examination is mandatory in cases of SCT and aims to screen for presacral lesions in early stages^5^. The non-compliance with these recommendations certainly compromises the treatment and safety of patients with SCT. The two centers differed on the performance of the rectal exam. 

In this study, SCT post-treatment sequelae were observed in 32% of patients, mainly urinary incontinence or retention, and tumor recurrence. The findings of this study were compatible with the data present in the literature, where urological sequelae are the most common^9^ ^,^ ^21^ ^,^ ^22^. The loss of follow-up in 20% of patients may underestimate the total number of cases that presented functional sequelae, in addition to survival analysis.

Recurrence is also present in follow-up studies and is commonly associated with incomplete resection of the coccyx or inadequate treatment of malignant cases, either with loss to follow-up or lack of neoadjuvant or adjuvant treatment, when these are indicated^14^ ^,^ ^23^. These findings reinforce the importance of a systematized treatment for all cases of SCT.

Regardless of the histopathological diagnosis, SCTs must be followed up for at least 5 years, with no maximum time for follow-up^5^. Because of the previously described possibility of relapses after long periods, sometimes even longer than 10 years, long-term follow-up is chosen^24^.

In the presence of malignant SCT, death is an expected outcome for cases of multiple recurrences. In general, malignant GCTs respond very well to chemotherapy, with high rates of survival and cure, even in cases of recurrence^5^ ^,^ ^8^. However, patients showing poor response to chemotherapy from the beginning of treatment, as well as several relapses, have a high mortality rate^23^. One of the deaths reported in this series fits this context. The second death was related to complications due to the presence of Patau’s syndrome^25^ ^-^ ^27^. 

The frequency of death in the series presented here may be mistaken as a consequence of the loss of follow-up of 20% of the sample because of the significant lack of follow-up in the non-oncology center.

In general, clinical protocols allow the standardization of procedures to achieve the best success rate in treatments. In cases of SCT, staging, collection of tumor markers, and specific imaging tests are fundamental to define the appropriate conduct for each case. An evaluation of the need for neoadjuvant treatment, as well as the surgical strategy to be used, are based on the information gathered through this initial assessment of the patients.

The adequate patients’ follow-up, as guided by the protocol, is also essential to achieve the highest rates of survival and cure, in the long term. Imaging scans, physical exams with rectal examination and follow-up of serum tumor markers allow the identification of possible recurrences, enabling an early approach.

Among the limitations of this study, we highlight its retrospective design and the small casuistic, readily justified by the rarity of the pathology studied. The cases are from the two largest reference centers in the state of Rio de Janeiro, making the study relevant within the context of rarity of this pathology.

## FINAL CONSIDERATIONS

Over the past decades, the development and inclusion of patients in research protocols have allowed a better understanding of many aspects related to all germ cell tumors. The collection of information from all possible cases is of key importance for the optimization of the approach, treatment, and follow-up of these patients.

The inclusion of the evaluation of the presence or absence of the coccyx in the sample sent to the pathologist should be discussed, and strongly suggested to become a standard topic in the histopathological analysis of SCT tumor.

It is important to emphasize that SCT is an oncologic pathology, although most cases present a histology considered benign. Therefore, all patients diagnosed with this pathology should preferably be treated according to the best available protocol, regardless of the center they are referred to^5^.
